# Supplementary material for: Changes in Wolf Occupancy and Feeding Habits in the Northern Apennines: Results of Long-Term Predator–Prey Monitoring
Source: Animals (Basel). 2024 Feb 27;14(5):735. doi: 10.3390/ani14050735 (PMC10931095; doi:10.3390/ani14050735)
Supplement: Supplementary file 1 [file animals-14-00735-s001.zip › animals-2853854-supplementary.pdf]

Changes in Wolf Occupancy and Feeding Habits in the Northern Apennines: Results of Long-Term Predator–Prey Monitoring

Elisa Torretta *, Anna Brangi and Alberto Meriggi

Department of Earth and Environmental Sciences, University of Pavia, Via Ferrata 1, 27100 Pavia, Italy;
anna.brangi@gmail.com (A.B.); alberto.meriggi@unipv.it (A.M.)
* Correspondence: torretta.elisa@gmail.com

Supplementary Material

Table S1 - Adequacy of sample size used to investigate the wolf diet in the Lombard Apennines (Northern Apennines, Italy). The sample size was adequate to represent the wolf diet. In fact, in every subsample the diversity curve reached an asymptote and the incremental change dropped below 1% with n < 10.

Mountainous zone	Upper-hill zone	Lower-hill zone
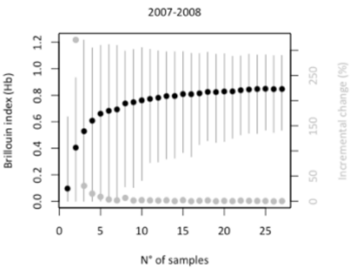		
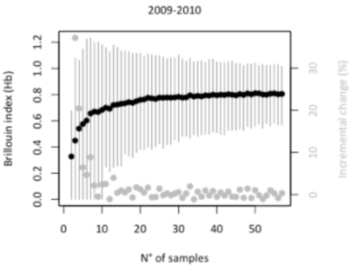		
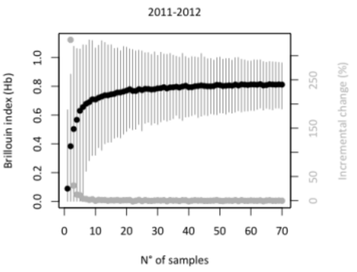		
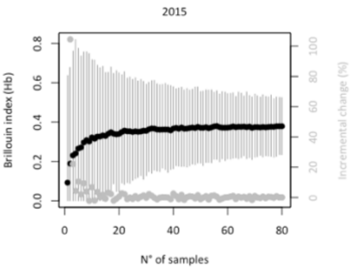	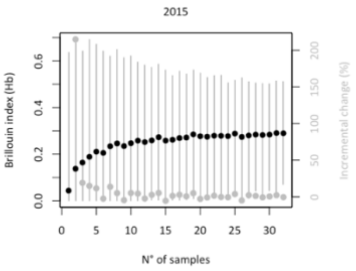	

Mountainous zone	Upper-hill zone	Lower-hill zone
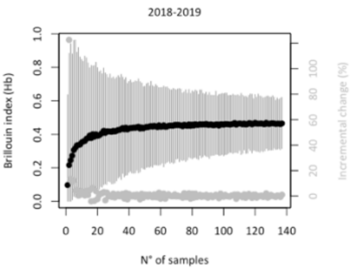	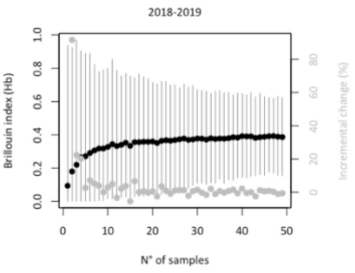	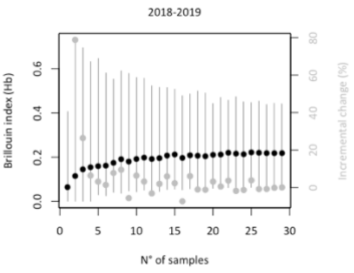
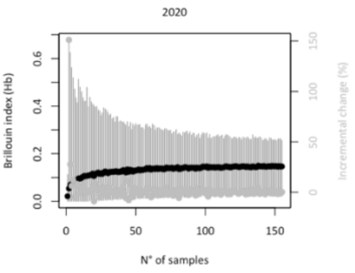	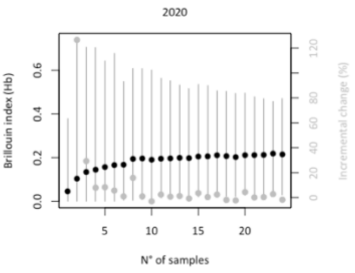	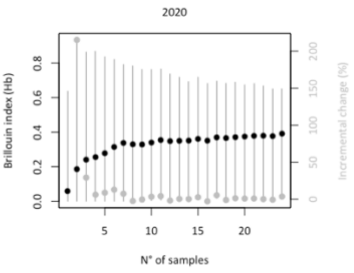
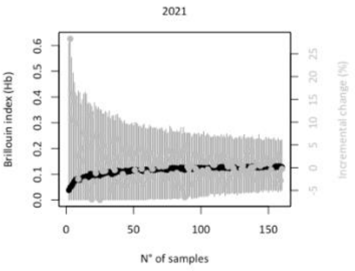	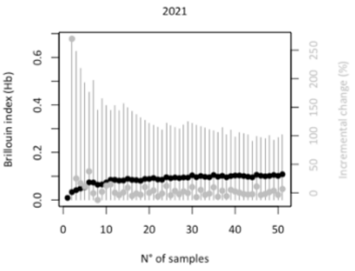	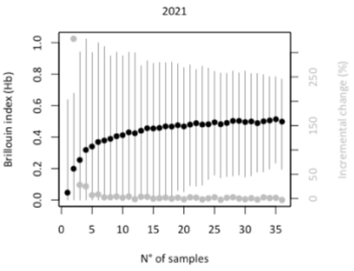

Table S2 - Details on the food habits of the wolf within the three zones of the Lombard Apennines (Northern Apennines, Italy).

Categories and species	Mountainous zone													
	2007-2008		2009-2010		2011-2012		2015		2018-2019		2020		2021	
	V%	SE	V%	SE	V%	SE	V%	SE	V%	SE	V%	SE	V%	SE
**Livestock**	**9.59**	**5.43**	**18.14**	**4.80**	**29.27**	**5.04**	**7.39**	**2.35**	**2.68**	**1.33**	**1.26**	**0.89**	**0.00**	**0.00**
Ovis aries	3.63	3.63	1.72	1.72	0.54	0.54	4.74	1.69	0.00	0.00	0.63	0.63	0.00	0.00
Capra hircus	2.33	2.33	6.49	3.03	12.51	3.63	1.42	1.24	0.72	0.72	0.00	0.00	0.00	0.00
Bos taurus	3.63	3.63	8.21	3.43	16.21	4.08	1.23	1.22	1.25	0.88	0.63	0.63	0.00	0.00
Equus caballus	0.00	0.00	1.72	1.72	0.00	0.00	0.00	0.00	0.72	0.72	0.00	0.00	0.00	0.00
**Wild ungulates**	**68.18**	**8.25**	**68.16**	**5.75**	**64.38**	**5.31**	**89.12**	**2.65**	**88.39**	**2.22**	**95.45**	**1.13**	**95.05**	**1.19**
Sus scrofa	55.88	8.92	25.51	5.43	31.9	5.38	25.54	4.36	9.83	2.45	5.57	1.74	10.94	2.40
Capreolus capreolus	5.04	3.84	39.43	6.17	12.6	3.54	61.12	4.94	67.83	3.72	85.46	2.58	74.93	3.20
Dama dama	7.26	5.03	0.00	0.00	1.4	1.40	2.45	1.72	7.25	2.15	3.16	1.40	6.74	1.97
Cervus elaphus	0.00	0.00	3.22	2.26	18.5	4.38	0.00	0.00	3.49	1.54	1.26	0.89	2.45	1.21
**Small mammals**	**0.57**	**0.57**	**3.71**	**2.42**	**3.02**	**1.97**	**1.61**	**1.25**	**3.20**	**1.42**	**0.63**	**0.63**	**0.39**	**0.39**
**Medium-sized mammals**	**16.85**	**7.03**	**5.16**	**2.92**	**1.62**	**1.41**	**0.67**	**0.51**	**1.71**	**1.04**	**0.00**	**0.00**	**0.98**	**0.66**
**Birds**	**0.00**	**0.00**	**0.00**	**0.00**	**0.00**	**0.00**	**0.00**	**0.00**	**0.00**	**0.00**	**0.00**	**0.00**	**0.61**	**0.61**
**Invertebrates**	**0.57**	**0.57**	**0.00**	**0.00**	**0.44**	**0.31**	**0.03**	**0.03**	**0.00**	**0.00**	**0.31**	**0.25**	**0.02**	**0.02**
**Fruits**	**1.33**	**0.80**	**0.00**	**0.00**	**0.00**	**0.00**	**0.00**	**0.00**	**2.60**	**0.57**	**0.40**	**0.25**	**0.24**	**0.24**
**Grasses**	**0.00**	**0.00**	**0.00**	**0.00**	**0.00**	**0.00**	**0.00**	**0.00**	**0.00**	**0.00**	**0.02**	**0.02**	**0.00**	**0.00**
**Garbage**	**0.00**	**0.00**	**0.00**	**0.00**	**0.00**	**0.00**	**0.00**	**0.00**	**0.05**	**0.03**	**0.00**	**0.00**	**0.00**	**0.00**

Categories and species	Upper-hill zone													
	2007-2008		2009-2010		2011-2012		2015		2018-2019		2020		2021	
	V%	SE	V%	V%	SE	V%	SE	V%	SE	V%	SE	SE	V%	SE
**Livestock**	**-**	**-**	**-**	**-**	**-**	**-**	**7.31**	**4.37**	**2.84**	**1.91**	**0.00**	**0.00**	**0.00**	**0.00**
Ovis aries	-	-	-	-	-	-	4.25	3.25	0.00	0.00	0.00	0.00	0.00	0.00
Capra hircus	-	-	-	-	-	-	3.06	3.06	0.32	0.32	0.00	0.00	0.00	0.00
Bos taurus	-	-	-	-	-	-	0.00	0.00	2.52	1.90	0.00	0.00	0.00	0.00
Equus caballus	-	-	-	-	-	-	0.00	0.00	0.00	0.00	0.00	0.00	0.00	0.00
**Wild ungulates**	**-**	**-**	**-**	**-**	**-**	**-**	**89.80**	**4.37**	**87.71**	**3.65**	**93.40**	**4.09**	**95.87**	**1.98**
Sus scrofa	-	-	-	-	-	-	25.78	7.30	25.20	5.89	28.6	9.29	16.16	4.49

Capreolus capreolus	-	-	-	-	-	-	57.89	8.33	52.44	6.67	52.6	10.10	73.95	5.35
Dama dama	-	-	-	-	-	-	6.12	4.26	4.00	2.80	12.2	6.76	5.76	3.26
Cervus elaphus	-	-	-	-	-	-	0.00	0.00	6.06	3.26	0.0	0.00	0.00	0.00
**Small mammals**	-	-	-	-	-	-	**0.00**	**0.00**	**0.00**	**0.00**	**4.08**	**4.08**	**0.00**	**0.00**
**Medium-sized mammals**	-	-	-	-	-	-	**0.48**	**0.48**	**2.00**	**2.00**	**0.00**	**0.00**	**1.92**	**1.92**
**Birds**	-	-	-	-	-	-	**0.00**	**0.00**	**0.00**	**0.00**	**0.00**	**0.00**	**0.00**	**0.00**
**Invertebrates**	-	-	-	-	-	-	**0.09**	**0.08**	**0.00**	**0.00**	**0.00**	**0.00**	**0.30**	**0.30**
**Fruits**	-	-	-	-	-	-	**0.02**	**0.02**	**4.42**	**1.80**	**1.33**	**0.65**	**0.00**	**0.00**
**Grasses**	-	-	-	-	-	-	**0.02**	**0.02**	**0.00**	**0.00**	**0.00**	**0.00**	**0.00**	**0.00**
**Garbage**	-	-	-	-	-	-	**0.00**	**0.00**	**0.00**	**0.00**	**0.00**	**0.00**	**0.00**	**0.00**

**Categories and species**	**Lower-hill zone**													
	**2007-2008**		**2009-2010**		**2011-2012**		**2015**		**2018-2019**		**2020**		**2021**	
	**V%**	**SE**	**V%**	**V%**	**SE**	**V%**	**SE**	**V%**	**SE**	**V%**	**SE**	**SE**	**V%**	**SE**
**Livestock**	-	-	-	-	-	-	-	-	**3.38**	**3.38**	**10.31**	**5.79**	**5.44**	**3.79**
Ovis aries	-	-	-	-	-	-	-	-	0.00	0.00	7.69	5.33	0.00	0.00
Capra hircus	-	-	-	-	-	-	-	-	3.38	3.38	2.62	2.63	0.00	0.00
Bos taurus	-	-	-	-	-	-	-	-	0.00	0.00	0.00	0.00	5.44	3.79
Equus caballus	-	-	-	-	-	-	-	-	0.00	0.00	0.00	0.00	0.00	0.00
**Wild ungulates**	-	-	-	-	-	-	-	-	**94.28**	**3.40**	**87.07**	**6.04**	**84.64**	**5.25**
Sus scrofa	-	-	-	-	-	-	-	-	17.0	6.99	24.6	8.02	10.97	4.87
Capreolus capreolus	-	-	-	-	-	-	-	-	63.8	8.75	50.2	9.82	68.23	7.02
Dama dama	-	-	-	-	-	-	-	-	13.5	6.39	12.2	6.76	5.44	3.79
Cervus elaphus	-	-	-	-	-	-	-	-	0.0	0.00	0.0	0.00	0.00	0.00
**Small mammals**	-	-	-	-	-	-	-	-	**0.00**	**0.00**	**0.00**	**0.00**	**0.00**	**0.00**
**Medium-sized mammals**	-	-	-	-	-	-	-	-	**0.00**	**0.00**	**0.65**	**0.65**	**5.44**	**3.79**
**Birds**	-	-	-	-	-	-	-	-	**0.00**	**0.00**	**0.00**	**0.00**	**0.00**	**0.00**
**Invertebrates**	-	-	-	-	-	-	-	-	**0.09**	**0.09**	**0.12**	**0.11**	**2.61**	**1.51**
**Fruits**	-	-	-	-	-	-	-	-	**1.74**	**0.54**	**0.38**	**0.17**	**0.00**	**0.00**
**Grasses**	-	-	-	-	-	-	-	-	**0.00**	**0.00**	**0.00**	**0.00**	**0.00**	**0.00**
**Garbage**	-	-	-	-	-	-	-	-	**0.00**	**0.00**	**0.00**	**0.00**	**0.00**	**0.00**
